# Effective long-term treatment with bevacizumab for relapsed glioblastoma: case report and review of the literature

**DOI:** 10.1186/2162-3619-3-29

**Published:** 2014-12-17

**Authors:** Katrin Schweneker, Christoph Clemm, Melanie Brügel, Michael Souvatzoglou, Mirjam Hermisson, Friederike Schmidt-Graf, Claus Zimmer, Christian Peschel, Philipp J Jost

**Affiliations:** III. Medizinische Klinik, München, Germany; Hämatologische Praxis, München, Germany; Institut für Diagnostische und Interventionelle Radiologie, München, Germany; Nuklearmedizinische Klinik und Poliklinik, München, Germany; Neurologische Klinik und Poliklinik, München, Germany; Abteilung für Diagnostische und Interventionelle Neuroradiologie, Klinikum rechts der Isar, Technische Universität München, 81675 München, Germany

**Keywords:** Relapsed glioblastoma, Bevacizumab, Pseudoprogression, ^18^ F-FET-PET/CT scan

## Abstract

Glioblastoma multiforme (GBM) is the most common malignant primary brain tumor in adults. Despite the use of optimized first-line therapy, GBM is still associated with a poor prognosis and an effective second-line therapy remains an important challenge in this patient population. In 2009, the US Food and Drug Administration (FDA) approved the monoclonal anti-VEGF-antibody bevacizumab for the treatment of relapsed GBM after two phase-II studies showed its efficacy and safety, alone or in combination with irinotecan, in relapsed GBM. In contrast, the European Medicines Agency (EMA) concluded from the same published data that a clear benefit in terms of overall survival was not shown and subsequently did not grant approval for bevacizumab in this setting. Here, we report on a 53-year old patient with relapsed GBM who was treated with bevacizumab as single agent. After three months, the tumor volume was reduced and the Karnofsky performance status was substantially improved compared to the baseline at the time of relapse. After continued long-term treatment for 26 months, the patient remains in an excellent general condition. Moreover, the measurement of the tumor volume using multiple imaging modalities shows a sustained treatment response. In conclusion, this case supports the notion that individual patients respond exceptionally well to treatment with anti-VEGF therapy and suggests that future trials are needed to better identify the patient population that responds to bevacizumab.

## Introduction

Glioblastoma multiforme (GBM) is the most common malignant primary brain tumor in adults and the 5-year survival rate is <5% in adults [1, 2]. According to the guidelines of the National Guideline Clearinghouse of the U.S. Department of Health & Human Services, the recommended therapy for a newly diagnosed GBM is surgical debulking provided that unacceptable neurologic deficit can be avoided, followed by adjuvant radiotherapy [3, 4]. Concomitant radiotherapy and temozolomide followed by adjuvant temozolomide for 6 courses have been shown to significantly prolong overall survival (OS) compared to radiotherapy alone [5, 6]. The median OS in patients treated with additional temozolomide is prolonged from 12.1 months to 14.6 months when compared to radiotherapy alone. In addition, the 2-year survival is improved from 10.4% with radiotherapy alone to 26.5% with radiotherapy and temozolomide [6]. Due to their invasive growth pattern, GBM have a high relapse rate [7]. According to the guidelines of the National Comprehensive Cancer Network (NCCN), the recommended therapy for a resectable locally relapsed GBM is resection with or without implantation of a carmustine wafer. However, there is no clearly defined standard therapy for relapsed unresectable GBM [8, 9]. In this case, the treatment decision depends on multiple parameters such as prior therapy, time to relapse, tumor grade, and performance status [8, 9]. Treatment options include repeated surgery, radiation, or chemotherapy [10]. Chemotherapeutic regimes include either monotherapy with lomustine (CCNU), platinum compounds, or temozolomide [9, 11, 12]. Alternatively, medically fit and healthy patients can be treated with polychemotherapy using regimens such as procarbacin/CCNU/vincristin (PCV) [13]. Two uncontrolled phase II studies showed that the monoclonal human anti-VEGF-antibody bevacizumab, alone or in combination with irinotecan, prolongs progression-free survival (PFS) and overall survival (OS) in patients with relapsed GBM compared to historical data [14, 15]. Based on this data, bevacizumab was approved by the US Food and Drug administration (FDA) for use in relapsed GBM. However, it is currently not approved by the European Medicines Agency (EMA) for relapsed GBM because of missing evidence that bevacizumab prolongs survival. In 2013, Johnson *et al*. published a population-based analysis in which they compared patients in the United States who eventually succumbed to GBM in 2006, 2008, and 2010 [16]. The difference in survival between 2008 and 2010 was significantly better for patients treated with bevacizumab [16]. This circumstantial evidence suggests that the improved median survival of GBM patients in the US might be due to the approval and use of bevacizumab, however a direct comparison of subpopulations is missing so far. Here we report on the successful long-term treatment of a relapsed GBM patient with bevacizumab as monotherapy for the last 26 months with an ongoing improvement in tumor volume and quality of life.

## Case report

**Figure 1:**
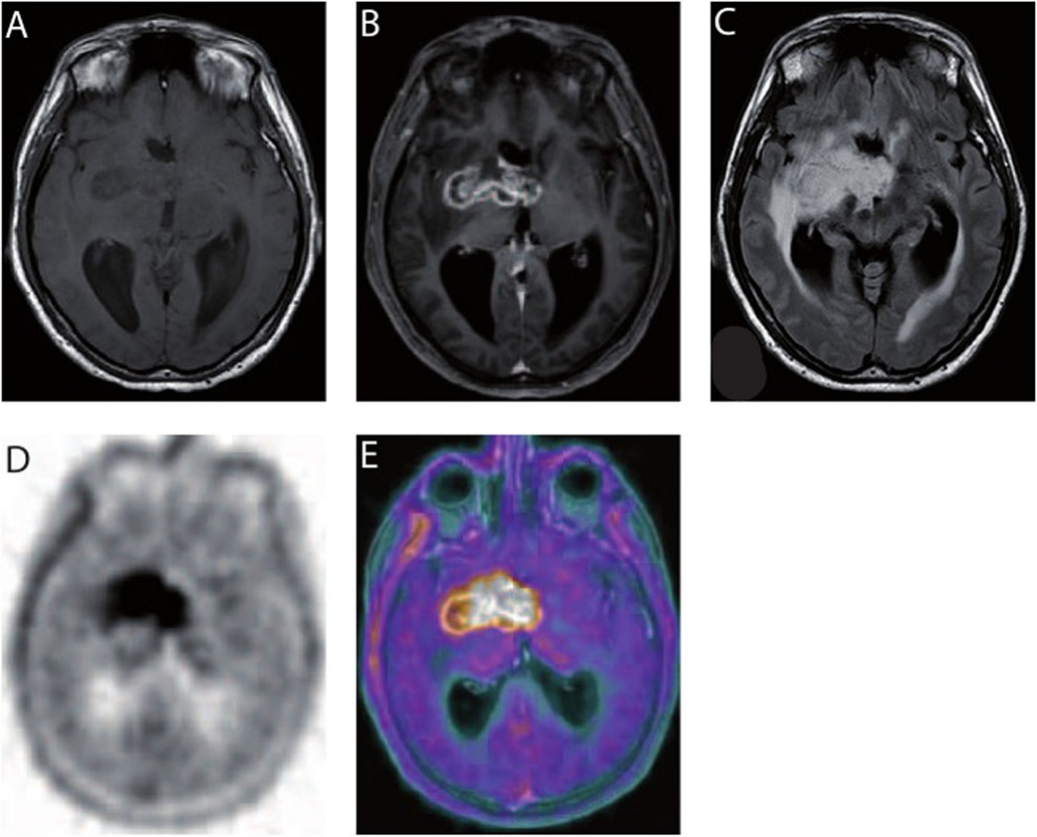
**Relapse of GBM after resection and radiochemotherapy. A** Native T1-weighted MRI shows a tumor involving the right basal ganglia. **B** Gadolinium-enhanced T1-weighted MRI shows strong peripheral and irregular enhancement of the tumor. **C** T2-weighted FLAIR demonstrates pronounced surrounding edema and mass effect. **D** In the PET/CT scan and **E** the fusion of PET and MRI, the lesion described in the MRI corresponds with focal increased ^18^ F-FET uptake exhibiting Tu/Bg ratio of 2.4.

**Figure 2:**
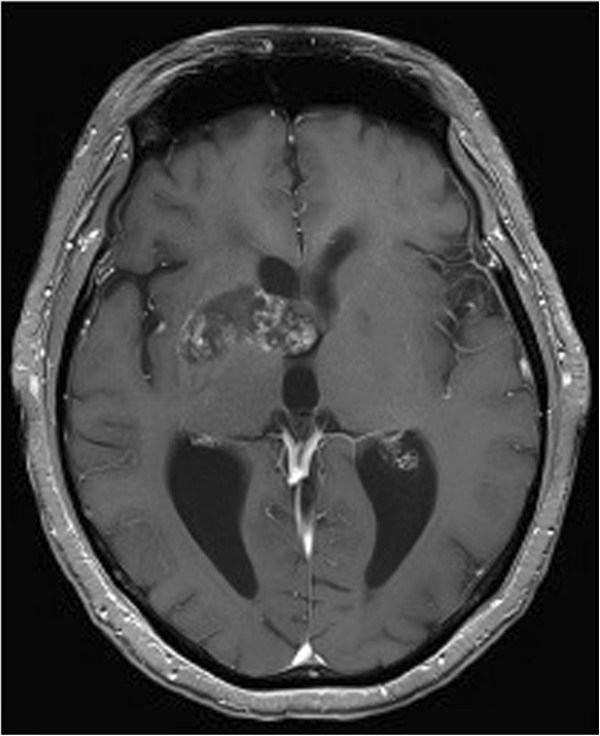
**GBM after three courses of bevacizumab.** Gadolinium-enhanced T1-weighted MRI shows a reduction in tumor size as well as decreasing and discontinuous tumor enhancement.

**Figure 3:**
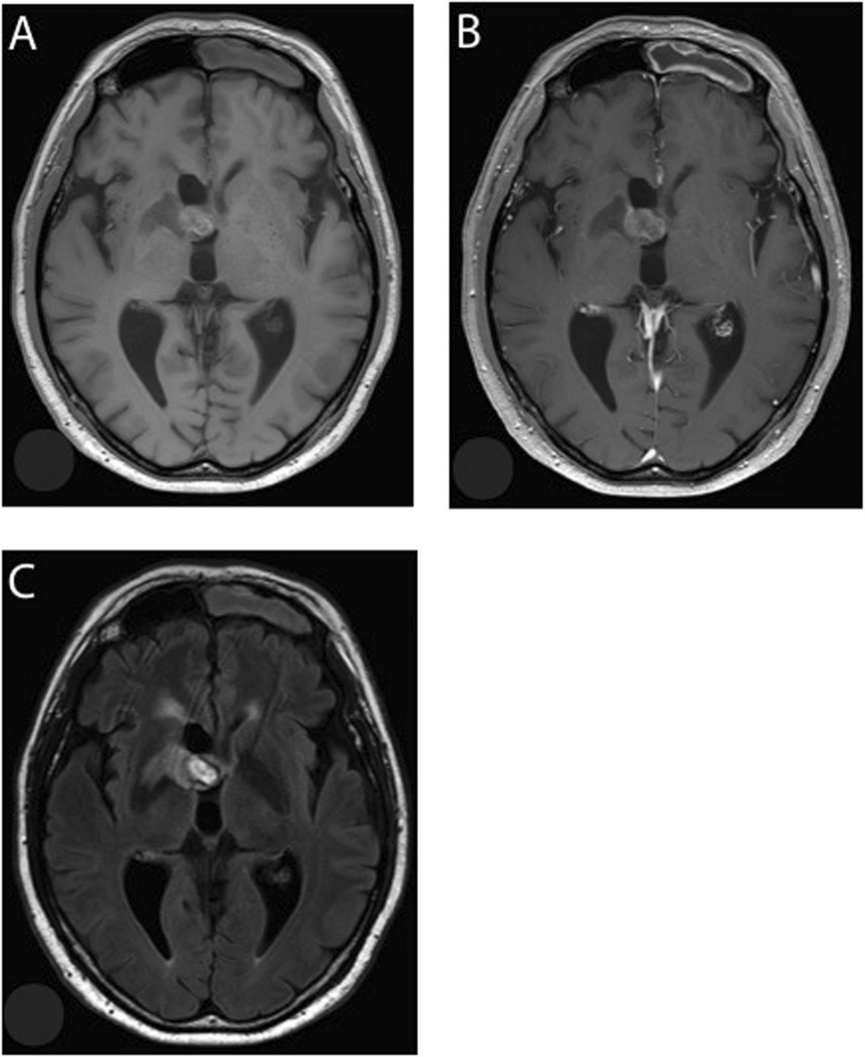
**Treatment response after 17 courses of bevacizumab in cMRI. A** Non-enhanced T1-weighted MRI reveals further decrease in lesion size. Hyperintense hemorrhagic changes are present within the medial portion of the tumor. **B** Gadolinium-enhanced T1-weighted MRI documents lack of contrast enhancement in the lateral portions of the tumor. Only a slight enhancement in addition to the hyperintense hemorrhagic changes in the medial portion of the tumor was observed. **C** T2-weighted FLAIR demonstrates a decrease of peritumoral edema.

**Figure 4:**
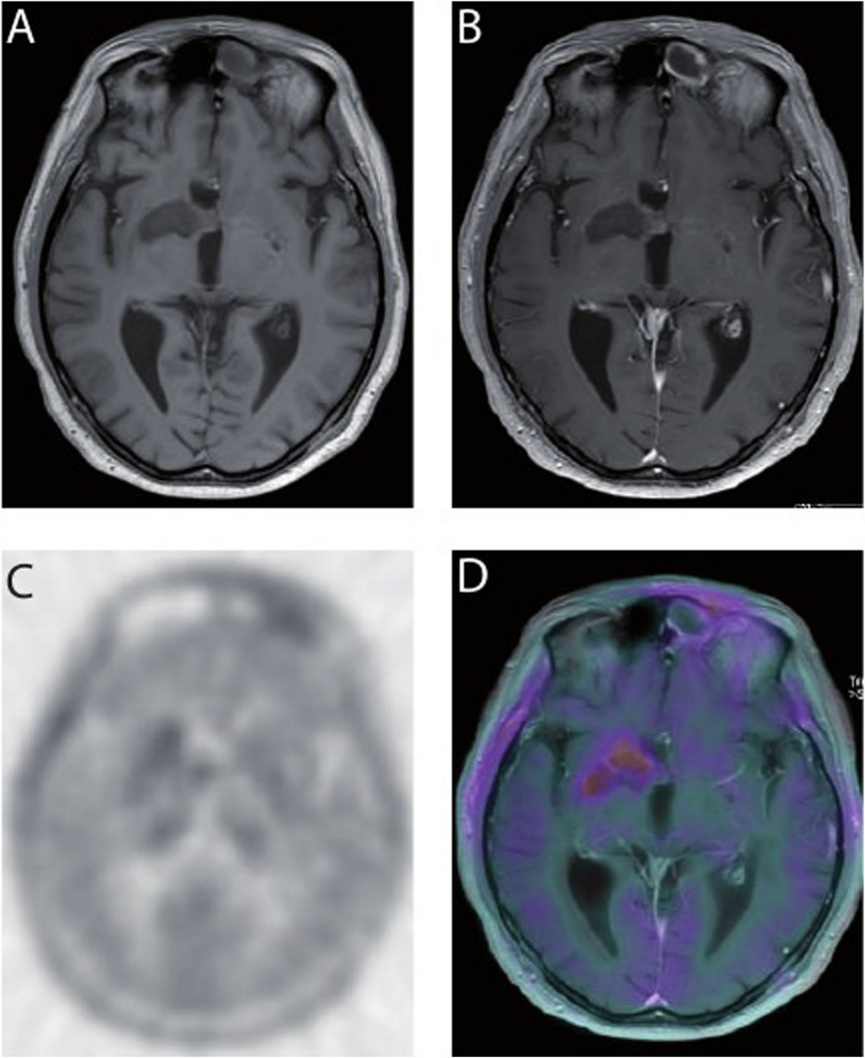
**Treatment response after 17 courses of bevacizumab in** ^**18**^ **F-FET-PET/CT and fusion of PET with MRI. A** The native and **B** the contrast-enhanced T1 cMRT scans show a decrease of the lesion initially described. **C** In the PET/CT and **D** the fusion of PET and MRI, the ^18^ F-FET uptake is slightly diffuse increased with Tu/Bg uptake ration of 1.3 consitent with reactive changes after treatment and arguing against vital tumor tissue.

In November 2011, a 53-year-old male patient presented to our clinics for chemoradiotherapy after resection of a cerebral lesion that was diagnosed as GBM WHO IV° by histopathology. An epigenetic silencing of O^6^-methylguanine-DNA methyltransferase (MGMT) by promoter methylation was not detected by real-time PCR of tumor DNA. Approximately six weeks after resection, the patient was started on standard combined chemotherapy and radiation (temozolomide 75 mg/m^2^/day and involved-field radiation at a total dose of 60 Gy in 2.0 Gy fractions over a 6-week period). The radiotherapy was well tolerated and the treatment with temozolomide was continued with 150 mg/m^2^ d1-5 in the first cycle followed by 200 mg/m^2^ d1-5 every 4 weeks. In April 2012, after three courses of temozolomide, the patient presented with intermittent disorientation and a worsened general condition. The native and Gadolinium-enhanced T1-weighted cerebral magnetic resonance imaging (cMRI) showed a tumor involving the right basal ganglia with strong peripheral and irregular contrast enhancement (Figure 1A and B). In the T2-weighted FLAIR a pronounced surrounding edema and mass effect was seen in the area of the basal ganglia (Figure 1C). Because of the clinical symptoms, the deteriorated general condition, and the unmethylated MGMT promoter status [17] these radiographic findings were interpreted as GBM relapse. A radiotherapy-induced pseudoprogression is not considered to cause the radiographic findings. To confirm this we performed ^18^ F-fluoroethyltyrosine-positron emission tomography/computer tomography (FET-PET/CT) scan (Figure 1D and E). The PET/CT (Figure 1D) showed a focally increased ^18^ F-FET uptake corresponding to the contrast enhancement described in cMRI, which was emphasized in the fusion of PET and MRI (Figure 1E). The mean tumoral uptake (Tu) was evaluated and the ratio to background (Bg) was calculated with Tu/Bg ratio of 2.4. This value exceeds the threshold of 1.6 thereby indicating an active malignancy [18]. Because of the localization of the relapsed GBM and the reduced general performance status, resection was deemed impossible. It was decided to treat the patient with anti-VEGF antibody (bevacizumab) monotherapy (Avastin®) in an off-label setting. Bevacizumab was given at a dose of 10 mg/kg body weight on d1 and d15 every four weeks. Concurrently, the palliative care team was involved in patient’s care because of the poor general performance status. After three courses, a reduction in tumor size from 51 × 21 × 25 mm to 39 × 17 × 17 mm was observed at Gadolinium-enhanced T1-weighted cMRI, and the degree of contrast enhancement within the tumor had decreased (Figure 2). On day one of the forth course of bevacizumab, our patient presented with dyspnoe at the emergency room and the suspected diagnosis of pulmonary embolism was confirmed by CT scan. Therapeutic anticoagulation was started using low molecular weight heparin. Bevacizumab was continued one week later at the initially used dosing regimen. Under low molecular weight heparin, there were no further thromboembolic events or other bevacizumab-related serious adverse reactions until to date. During ongoing therapy with bevacizumab, the patient’s Karnofsky performance status improved steadily from 50% at the beginning of treatment with bevacizumab to 80% after 6 months and the palliative care team stopped patient’s care. Concomitant corticosteroid therapy was reduced continuously and finally stopped. The last cMRI performed after 17 courses of bevacizumab showed a very good partial response to bevacizumab therapy with a tumor size of 35 × 15 × 15 mm in the unenhanced T1-weighted sequences (Figure 3A) and a lack of contrast enhancement in the lateral portions of the tumor in the Gadolinium-enhanced T1-weighted sequences (Figure 3B). T2-weighted FLAIR demonstrated a significant decrease of peritumoral edema (Figure 3C). A very low level of ^18^ F-FET tracer uptake was noted (Figure 4), which was substantially lower than the tracer uptake observed before treatment (Figure 1D and E). Moreover, Tu/Bg ratio in the post treatment PET/CT scan was 1.3 consistent with reactive changes after treatment (Figure 4). After the excellent treatment response observed under bevacizumab treatment, we evaluated to stop monotherapy with bevacizumab. However, since remaining active tumor cannot be fully excluded by the imaging modalities used, we decided together with the interdisciplinary tumor board to further extend the treatment.

26 months after the diagnosis of relapsed GBM, our patient remains in an excellent general condition with a Karnofsky performance status of 100% without further adverse side effects to the current therapy. Under ongoing treatment with bevacizumab the patient is able to live a self-reliant, socially active life.

## Discussion

Despite therapeutic advances, the prognosis for patients with GBM is still poor [19]. Nevertheless, a small fraction of patients with GBM (3–5%) survive longer that 36 months and they are referred to as long-term survivors [2]. Young age, female gender, higher preoperative Karnofsky performance status, unilateral tumor, gross total resection, and adjuvant radiochemotherapy are associated with prolonged survival [20]. Furthermore, there are some molecular markers, e.g. p53 mutation, MGMT promoter methylation, or isocitrate dehydrogenase 1 (IDH1) mutations that are frequently found in long-term survivors [20]. Single amino acid mutations in IDH1 can lead to the loss of regular enzyme function [21]. The mutated IDH1 converts α-ketoglutarate to the oncometabolite 2-hydroxyglutarate (2-HG), which causes genome-wide epigenetic changes in human gliomas [22]. Patients harboring mutated IDH1 and corresponding epigenetic changes have a better prognosis than patients diagnosed with GBM with wild-type IDH1 [21, 23]. This is exemplified by the fact that treatment with bevacizumab as single agent in patients with relapsed GBM and IDH1 mutation results in a significant better PFS and OS compared to patients without IDH1 mutation (PFS 3.23 *vs.* 1.37 months, *p* = 0.05; and OS 10.16 *vs.* 4.90 months, *p* = 0.09) [24].

The MGMT promoter methylation is known to play an important role in the cellular response to alkylating agents such as temozolomide [5, 25]. Significantly more long-term survivors harbor MGMT promoter methylation compared to patients that survive shorter than 18 months [20]. Furthermore, Capdevila and colleagues showed, that patients with methylated MGMT promoter status derive greater benefit from neoadjuvant temozolomide treatment than those without methylated MGMT promoter status [26]. Concerning bevacizumab, a previously published single-arm study showed that patients with a relapsed GBM treated with the combination of bevacizumab and fotemustin had an improved PFS when the MGMT promoter was methylated [27], however the difference to historical controls was not statistically significant. An alternative single-arm study used bevacizumab in combination with temozolomide after radiotherapy in newly diagnosed GBM patients [28]. In this study, patients with MGMT promoter methylation had an improved median OS and PFS compared to patients without MGMT promoter methylation [28]. The patients of the MGMT unmethylated group appeared to have a poor outcome, possibly caused by the reduced effectiveness of temozolomide.

Since the approval of bevacizumab for relapsed GBM by the FDA, there is an ongoing attempt to improve the anti-tumor effect of bevacizumab using combination therapies. Several studies have analyzed the combination of bevacizumab with various chemotherapeutic agents that are recommended as second-line therapy for relapsed unresectable GBM. In these studies, bevacizumab was combined with irinotecan [14, 29, 30], oral etoposide [31], temozolomide [32], carboplatin and etoposide [33], or carboplatin and irinotecan [34]. All these combination therapies were effective treatments for relapsed GBM, but did not improve the outcome over the level observed with bevacizumab alone.

The presented case shows that monotherapy with bevacizumab can substantially prolong survival and improve quality of life in patients with relapsed GBM. This argues that a better understanding of the clinical or molecular parameters that define the patient population that will profit from bevacizumab monotherapy is urgently needed. At this point, it is not clear whether a definite correlation exists between improved PFS and OS in patients treated with bevacizumab as monotherapy and their MGMT methylation status. The patient’s current survival time of 26 months from start of monotherapy with bevacizumab after relapsed GBM exceeds all expectations and surpasses to our knowledge survival rates of other studies or reports. The patient remains in an excellent general condition under ongoing treatment with bevacizumab. At this point, we have no explanation for this successful course, particularly taking into consideration the reduced Karnofsky performance status of 50% at time of relapse, male gender and unmethylated MGMT promoter as negative prognostic survival markers. Therefore, additional clinical trials and an improved molecular characterization of these cases might help to identify patient populations that will likely benefit from an anti-VEGF therapy.

## References

[1] Dolecek TA, Propp JM, Stroup NE, Kruchko C (2012). CBTRUS statistical report: primary brain and central nervous system tumors diagnosed in the United States in 2005-2009. Neuro Oncol.

[2] Krex D, Klink B, Hartmann C, von Deimling A, Pietsch T, Simon M, Sabel M, Steinbach JP, Heese O, Reifenberger G (2007). Long-term survival with glioblastoma multiforme. Brain.

[3] Kristiansen K, Hagen S, Kollevold T, Torvik A, Holme I, Nesbakken R, Hatlevoll R, Lindgren M, Brun A, Lindgren S (1981). Combined modality therapy of operated astrocytomas grade III and IV. Confirmation of the value of postoperative irradiation and lack of potentiation of bleomycin on survival time: a prospective multicenter trial of the Scandinavian Glioblastoma Study Group. Cancer.

[4] Walker MD, Green SB, Byar DP, Alexander E, Batzdorf U, Brooks WH, Hunt WE, MacCarty CS, Mahaley MS, Mealey J (1980). Randomized comparisons of radiotherapy and nitrosoureas for the treatment of malignant glioma after surgery. N Engl J Med.

[5] Stupp R, Hegi ME, Mason WP, van den Bent MJ, Taphoorn MJ, Janzer RC, Ludwin SK, Allgeier A, Fisher B, Belanger K (2009). Effects of radiotherapy with concomitant and adjuvant temozolomide versus radiotherapy alone on survival in glioblastoma in a randomised phase III study: 5-year analysis of the EORTC-NCIC trial. Lancet Oncol.

[6] Stupp R, Mason WP, van den Bent MJ, Weller M, Fisher B, Taphoorn MJ, Belanger K, Brandes AA, Marosi C, Bogdahn U (2005). Radiotherapy plus concomitant and adjuvant temozolomide for glioblastoma. N Engl J Med.

[7] Ohgaki H, Kleihues P (2007). Genetic pathways to primary and secondary glioblastoma. Am J Pathol.

[8] Stupp R, Roila F (2009). Malignant glioma: ESMO clinical recommendations for diagnosis, treatment and follow-up. Ann Oncol.

[9] Weller M, Cloughesy T, Perry JR, Wick W (2013). Standards of care for treatment of recurrent glioblastoma–are we there yet?. Neuro Oncol.

[10] Stupp R, Hegi ME, van den Bent MJ, Mason WP, Weller M, Mirimanoff RO, Cairncross JG (2006). Changing paradigms–an update on the multidisciplinary management of malignant glioma. Oncologist.

[11] Scoccianti S, Detti B, Sardaro A, Iannalfi A, Meattini I, Leonulli BG, Borghesi S, Martinelli F, Bordi L, Ammannati F, Biti G (2008). Second-line chemotherapy with fotemustine in temozolomide-pretreated patients with relapsing glioblastoma: a single institution experience. Anticancer Drugs.

[12] Brandes AA, Tosoni A, Franceschi E, Blatt V, Santoro A, Faedi M, Amista P, Gardiman M, Labianca R, Bianchini C (2009). Fotemustine as second-line treatment for recurrent or progressive glioblastoma after concomitant and/or adjuvant temozolomide: a phase II trial of Gruppo Italiano Cooperativo di Neuro-Oncologia (GICNO). Cancer Chemother Pharmacol.

[13] Cairncross G, Macdonald D, Ludwin S, Lee D, Cascino T, Buckner J, Fulton D, Dropcho E, Stewart D, Schold C (1994). Chemotherapy for anaplastic oligodendroglioma. National Cancer Institute of Canada Clinical Trials Group. J Clin Oncol.

[14] Friedman HS, Prados MD, Wen PY, Mikkelsen T, Schiff D, Abrey LE, Yung WK, Paleologos N, Nicholas MK, Jensen R (2009). Bevacizumab alone and in combination with irinotecan in recurrent glioblastoma. J Clin Oncol.

[15] Kreisl TN, Kim L, Moore K, Duic P, Royce C, Stroud I, Garren N, Mackey M, Butman JA, Camphausen K (2009). Phase II trial of single-agent bevacizumab followed by bevacizumab plus irinotecan at tumor progression in recurrent glioblastoma. J Clin Oncol.

[16] Johnson DR, Leeper HE, Uhm JH (2013). Glioblastoma survival in the United States improved after Food and Drug Administration approval of bevacizumab: a population-based analysis. Cancer.

[17] Brandes AA, Franceschi E, Tosoni A, Blatt V, Pession A, Tallini G, Bertorelle R, Bartolini S, Calbucci F, Andreoli A (2008). MGMT promoter methylation status can predict the incidence and outcome of pseudoprogression after concomitant radiochemotherapy in newly diagnosed glioblastoma patients. J Clin Oncol.

[18] Dunet V, Rossier C, Buck A, Stupp R, Prior JO (2012). Performance of 18F-fluoro-ethyl-tyrosine (18F-FET) PET for the differential diagnosis of primary brain tumor: a systematic review and Metaanalysis. J Nucl Med.

[19] Goodenberger ML, Jenkins RB (2012). Genetics of adult glioma. Cancer Genet.

[20] Zhang GB, Cui XL, Sui DL, Ren XH, Zhang Z, Wang ZC, Lin S (2013). Differential molecular genetic analysis in glioblastoma multiforme of long- and short-term survivors: a clinical study in Chinese patients. J Neurooncol.

[21] Yan H, Parsons DW, Jin G, McLendon R, Rasheed BA, Yuan W, Kos I, Batinic-Haberle I, Jones S, Riggins GJ (2009). IDH1 and IDH2 mutations in gliomas. N Engl J Med.

[22] Turcan S, Rohle D, Goenka A, Walsh LA, Fang F, Yilmaz E, Campos C, Fabius AW, Lu C, Ward PS (2012). IDH1 mutation is sufficient to establish the glioma hypermethylator phenotype. Nature.

[23] Sanson M, Marie Y, Paris S, Idbaih A, Laffaire J, Ducray F, El Hallani S, Boisselier B, Mokhtari K, Hoang-Xuan K, Delattre JY (2009). Isocitrate dehydrogenase 1 codon 132 mutation is an important prognostic biomarker in gliomas. J Clin Oncol.

[24] Lv S, Teugels E, Sadones J, Quartier E, Huylebrouck M, DUF S, LEM M, DEW O, Salmon I, Michotte A (2011). Correlation between IDH1 gene mutation status and survival of patients treated for recurrent glioma. Anticancer Res.

[25] Hegi ME, Diserens AC, Gorlia T, Hamou MF, de Tribolet N, Weller M, Kros JM, Hainfellner JA, Mason W, Mariani L (2005). MGMT gene silencing and benefit from temozolomide in glioblastoma. N Engl J Med.

[26] Capdevila L, Cros S, Ramirez JL, Sanz C, Carrato C, Romeo M, Etxaniz O, Hostalot C, Massuet A, Cuadra JL (2014). Neoadjuvant cisplatin plus temozolomide versus standard treatment in patients with unresectable glioblastoma or anaplastic astrocytoma: a differential effect of MGMT methylation. J Neurooncol.

[27] Soffietti R, Trevisan E, Bertero L, Cassoni P, Morra I, Fabrini MG, Pasqualetti F, Lolli I, Castiglione A, Ciccone G, Ruda R (2014). Bevacizumab and fotemustine for recurrent glioblastoma: a phase II study of AINO (Italian Association of Neuro-Oncology). J Neurooncol.

[28] Lai A, Tran A, Nghiemphu PL, Pope WB, Solis OE, Selch M, Filka E, Yong WH, Mischel PS, Liau LM (2011). Phase II study of bevacizumab plus temozolomide during and after radiation therapy for patients with newly diagnosed glioblastoma multiforme. J Clin Oncol.

[29] Vredenburgh JJ, Desjardins A, Herndon JE, Dowell JM, Reardon DA, Quinn JA, Rich JN, Sathornsumetee S, Gururangan S, Wagner M (2007). Phase II trial of bevacizumab and irinotecan in recurrent malignant glioma. Clin Cancer Res.

[30] Vredenburgh JJ, Desjardins A, Herndon JE, Marcello J, Reardon DA, Quinn JA, Rich JN, Sathornsumetee S, Gururangan S, Sampson J (2007). Bevacizumab plus irinotecan in recurrent glioblastoma multiforme. J Clin Oncol.

[31] Reardon DA, Desjardins A, Vredenburgh JJ, Gururangan S, Sampson JH, Sathornsumetee S, McLendon RE, Herndon JE, Marcello JE, Norfleet J (2009). Metronomic chemotherapy with daily, oral etoposide plus bevacizumab for recurrent malignant glioma: a phase II study. Br J Cancer.

[32] Desjardins A, Reardon DA, Coan A, Marcello J, Herndon JE, Bailey L, Peters KB, Friedman HS, Vredenburgh JJ (2012). Bevacizumab and daily temozolomide for recurrent glioblastoma. Cancer.

[33] Francesconi AB, Dupre S, Matos M, Martin D, Hughes BG, Wyld DK, Lickliter JD (2012). Carboplatin and etoposide combined with bevacizumab for the treatment of recurrent glioblastoma multiforme. J Clin Neurosci.

[34] Reardon DA, Desjardins A, Peters KB, Gururangan S, Sampson JH, McLendon RE, Herndon JE, Bulusu A, Threatt S, Friedman AH (2012). Phase II study of carboplatin, irinotecan, and bevacizumab for bevacizumab naive, recurrent glioblastoma. J Neurooncol.

